# αT-Catenin Is a Constitutive Actin-binding α-Catenin That Directly Couples the Cadherin·Catenin Complex to Actin Filaments*

**DOI:** 10.1074/jbc.M116.735423

**Published:** 2016-05-26

**Authors:** Emily D. Wickline, Ian W. Dale, Chelsea D. Merkel, Jonathon A. Heier, Donna B. Stolz, Adam V. Kwiatkowski

**Affiliations:** From the Department of Cell Biology, University of Pittsburgh School of Medicine, Pittsburgh, Pennsylvania 15261

**Keywords:** actin, adherens junction, alpha-catenin (α-catenin), cadherin, cardiomyocyte

## Abstract

α-Catenin is the primary link between the cadherin·catenin complex and the actin cytoskeleton. Mammalian αE-catenin is allosterically regulated: the monomer binds the β-catenin·cadherin complex, whereas the homodimer does not bind β-catenin but interacts with F-actin. As part of the cadherin·catenin complex, αE-catenin requires force to bind F-actin strongly. It is not known whether these properties are conserved across the mammalian α-catenin family. Here we show that αT (testes)-catenin, a protein unique to amniotes that is expressed predominantly in the heart, is a constitutive actin-binding α-catenin. We demonstrate that αT-catenin is primarily a monomer in solution and that αT-catenin monomer binds F-actin in cosedimentation assays as strongly as αE-catenin homodimer. The β-catenin·αT-catenin heterocomplex also binds F-actin with high affinity unlike the β-catenin·αE-catenin complex, indicating that αT-catenin can directly link the cadherin·catenin complex to the actin cytoskeleton. Finally, we show that a mutation in αT-catenin linked to arrhythmogenic right ventricular cardiomyopathy, V94D, promotes homodimerization, blocks β-catenin binding, and in cardiomyocytes disrupts localization at cell-cell contacts. Together, our data demonstrate that αT-catenin is a constitutively active actin-binding protein that can physically couple the cadherin·catenin complex to F-actin in the absence of tension. We speculate that these properties are optimized to meet the demands of cardiomyocyte adhesion.

## Introduction

The adherens junction (AJ)^2^ mechanically couples the actin cytoskeletons of adjacent cells to establish and maintain intercellular adhesion (1–3). The core of the AJ is the cadherin·catenin complex (4). Classical cadherins are single pass transmembrane proteins with an extracellular domain that mediates calcium-dependent homotypic interactions (5). The adhesive properties of classical cadherins are driven by the recruitment of cytosolic catenin proteins to the cadherin tail: p120-catenin binds to the juxtamembrane domain, and β-catenin binds to the distal part of the tail (6). β-Catenin, in turn, recruits α-catenin to the cadherin·catenin complex (7, 8). α-Catenin is a filamentous actin (F-actin)-binding protein and the primary link between the AJ and the actin cytoskeleton (9–12).

In mammals, αE (epithelial)-catenin is allosterically regulated: the monomer binds the β-catenin·cadherin complex, whereas the homodimer does not bind β-catenin but interacts with F-actin (9, 10). β-Catenin binding to αE-catenin sterically hinders F-actin binding (8, 13), explaining how αE-catenin as part of the cadherin·catenin complex has a weak affinity for F-actin. More recently, it was shown that the cadherin·catenin complex binds strongly to F-actin under force, indicating that the αE-catenin-actin interface is dynamically regulated by tension (12). In addition, evidence suggests that tension can regulate αE-catenin conformation: actomyosin-generated force stretches the middle (M) domain to reveal binding sites for cytoskeletal proteins such as vinculin (14–18). Thus, αE-catenin is a dynamic and multifunctional protein regulated by tension.

α-Catenin functions in adhesion and mechanical signaling must be integrated in all tissues. In cardiomyocytes, the AJ functions with the desmosome to physically link opposing cells in a specialized adhesive structure called the intercalated disc (ICD) (19). Contractile forces place physical demands on heart junctional complexes: not only must they withstand repeated cycles of force, but tension-sensing proteins within these complexes must be “tuned” to regulate signaling and maintain homeostasis (20). Two α-catenin proteins are expressed in the mammalian heart, αE-catenin and αT (testes)-catenin (21–23). In contrast to the widely studied and well defined mammalian αE-catenin, little is known about αT-catenin, a protein unique to amniotes that is expressed predominantly in the heart and testes (22, 24). αT-Catenin is expressed in cardiomyocytes where it localizes to the ICD, and genetic ablation of αT-catenin in mice causes dilated cardiomyopathy (22, 23, 25). Notably, two mutations in αT-catenin have been linked to arrhythmogenic right ventricular cardiomyopathy (ARVC): an amino acid (aa) change in the N terminus (valine to aspartic acid, V94D) and deletion of one aa in the C-terminal ABD (loss of a leucine, L765del) (26). However, the molecular properties of αT-catenin are undefined, and how these mutations affect αT-catenin function in cardiomyocytes remains unclear.

Here we show that αT-catenin is a constitutive actin-binding α-catenin that can directly couple the AJ to the actin cytoskeleton. Our data also reveal that the V94D mutation linked to ARVC alters αT-catenin dimerization potential to disrupt β-catenin binding and cellular localization. We postulate that αT-catenin protein conformation and ligand binding properties are tuned to meet the specific demands of cardiomyocyte adhesion.

## Results

### 

#### 

##### αT-Catenin Domain Stability Differs from αE-Catenin

**FIGURE 1.**
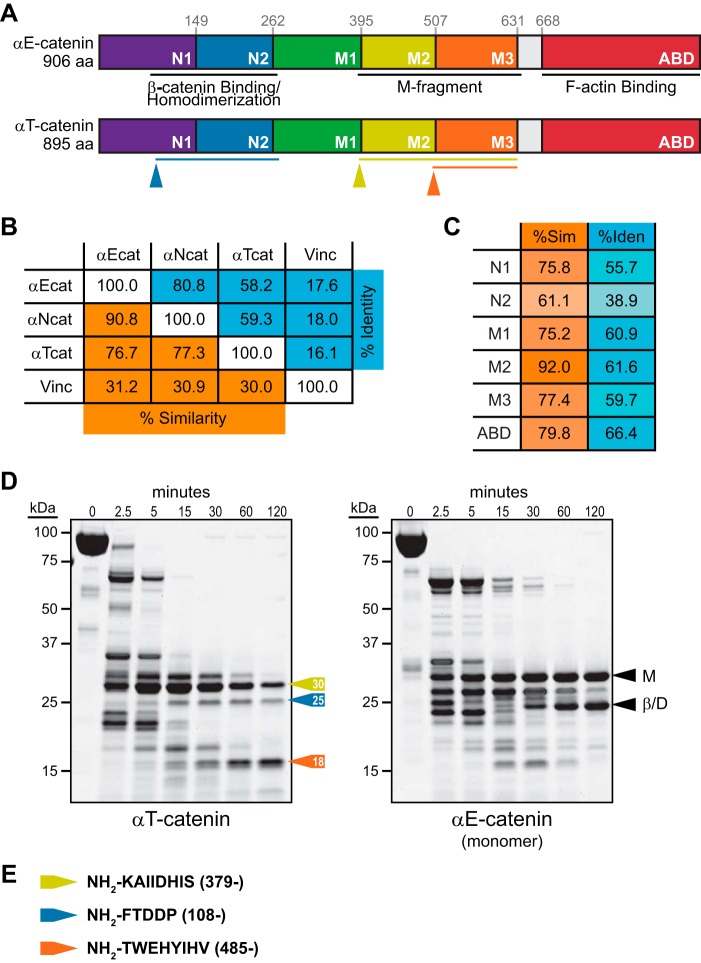
**αT-Catenin domain organization.** *A*, αE-catenin is composed of five four-helix bundles, a linker region, and a five-helix bundle tail. Domain amino acid boundaries are marked. The two N-terminal four-helix bundles (N1 and N2) bind β-catenin and mediate homodimerization (the protease-resistant region is *underlined*). The middle region contains three four-helix bundles (M1–M3; the protease-resistant M fragment is *underlined*). The C-terminal domain binds F-actin (ABD). αT-Catenin possesses a similar domain organization based on sequence homology. Trypsin-resistant fragments (from *D*) are shown as *color-coded lines* below αT-catenin. *B*, percent identity (*blue*) and percent similarity (*orange*) among *M. musculus* αE-catenin (α*Ecat*), αN-catenin (α*Ncat*), αT-catenin (α*Tcat*), and vinculin (*Vinc*). *C*, percent identity (%*Iden*) (*blue*) and percent similarity (%*Sim*) (*orange*) between *M. musculus* αE-catenin and αT-catenin domains. *D*, limited proteolysis of recombinant αT-catenin (*left*) and αE-catenin monomer (*right*). A Coomassie-stained SDS-polyacrylamide gel is shown for proteins incubated for 0, 2.5, 5, 15, 30, 60, and 120 min at room temperature in 0.05 mg/ml trypsin. M-fragment (*M*; aa 385–651) and β-catenin/dimerization (β*/D*; aa 82–287) fragments in αE-catenin are marked with *black arrows*. Stable αT-catenin fragments of 30 (*yellow*), 25 (*blue*), and 18 kDa (*orange*) are noted with *colored arrows. E*, Edman sequencing results of limited proteolysis fragments. Protein fragments are mapped on the full-length sequence (*A*) as color-coded lines.

Structural studies of αE-catenin have revealed that the protein is a series of helical bundles (7, 8, 13, 27, 28). The N-terminal (N) domain consists of two four-helix bundles (Fig. 1*A*, N1 and N2), binds β-catenin, and mediates homodimerization (7). The M region is composed of three four-helix bundles (Fig. 1*A*, M1–M3) and binds vinculin in response to mechanical force (14–17, 29, 30). A small linker region connects the C-terminal five-helix actin-binding domain (ABD) to the M region (Fig. 1*A*). We compared the amino acid sequence of *Mus musculus* αT-catenin with *M. musculus* αE-catenin and *M. musculus* αN-catenin. αT-Catenin is 58% identical and 77% similar to αE-catenin; likewise, it is 59% identical and 77% similar to αN-catenin (Fig. 1*B*). αE-Catenin and αN-catenin are 81% identical and 91% similar, making αT-catenin the most divergent of the mammalian family. We then analyzed sequence homology across domains between αT-catenin and αE-catenin (Fig. 1*C*). The region with the lowest degree of homology is N2 (39% identical and 61% similar), whereas the region with the highest degree of homology is M2 (62% identical and 92% similar).

We then questioned whether sequence differences affected domain organization in αT-catenin. We purified recombinant *M. musculus* αT-catenin and *M. musculus* αE-catenin from *Escherichia coli* and used limited trypsin proteolysis to examine domain organization. As shown previously (31, 32), tryptic digestion of αE-catenin monomer revealed two stable fragments: the modulation domain (aa 385–651) and the β-catenin-binding/homodimerization domain (aa 82–287) (Fig. 1*D*). Tryptic digestion of αT-catenin revealed three stable fragments at 30, 25, and 18 kDa (Fig. 1*D*). N-terminal sequencing revealed that the 30-kDa fragment started at aa 379 and contained bundles M2 and M3 (Fig. 1*D*). The entire M2-M3 region forms a protease-resistant fragment in mouse αE-catenin (Fig. 1*D*) (10, 31, 33) and fish αE-catenin (32). Notably, the 18-kDa fragment started at aa 485, near the end of domain M2, and contained the entire M3 domain. This suggests that, unlike αE-catenin, the αT-catenin M2-M3 region exists in a more open, protease-sensitive state. Finally, the 25-kDa fragment started at aa 108, similar to the dimerization/β-catenin-binding domain in αE-catenin (aa 82–287), although this fragment, similar to M2-M3, was markedly less protease-resistant than in αE-catenin. We conclude that the conformation of αT-catenin is similar to αE-catenin but with differences in the stability of both N-terminal and middle domains that could impact function.

##### αT-Catenin Is a Monomer in Solution

**FIGURE 2.**
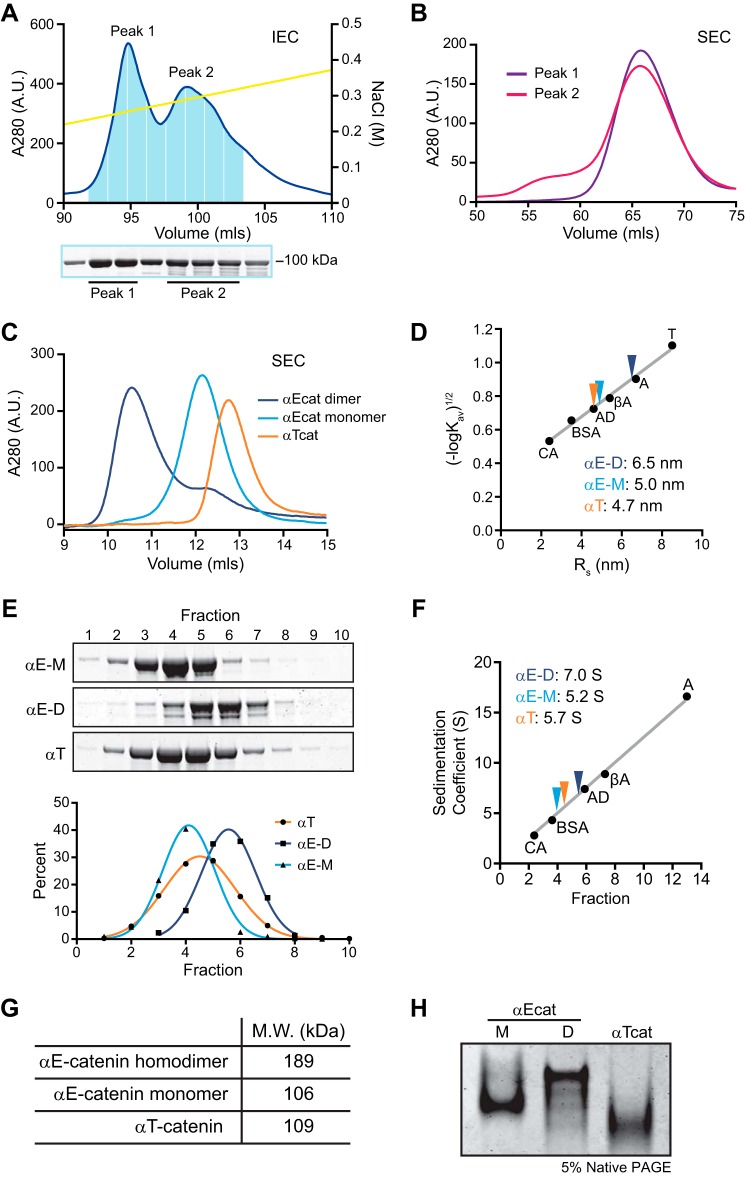
**αT-Catenin is a compact monomer.** *A*, Mono Q anion exchange chromatography of recombinant αT-catenin (*top*) and Coomassie-stained SDS-PAGE of fractions (*bottom*). *B*, S200 SEC of αT-catenin Mono Q peak fractions. *C*, analytical S200 SEC of recombinant αE-catenin homodimer, αE-catenin monomer, and αT-catenin. Elution profiles were used to calculate *K*_av_. *D*, Stokes radii of αE-catenin homodimer, αE-catenin monomer, and αT-catenin. *K*_av_ was calculated for standard proteins carbonic anhydrase (*CA*; *R_S_* = 2.4 nm), BSA (*R_S_* = 3.5 nm), alcohol dehydrogenase (*AD*; *R_S_* = 4.6 nm), β-amylase (β*A*; *R_S_* = 5.4 nm), apoferritin (*A*; *R_S_* = 6.7 nm), and thyroglobulin (*T*; *R_S_* = 8.5 nm). A standard curve was created by plotting (−logK_av_)^1/2^ *versus R_S_*. αE-Catenin homodimer, αE-catenin monomer, and αT-catenin *R_S_* values were determined from the standard curve. *E*, sucrose gradient sedimentation of αE-catenin monomer (α*E-M*), αE-catenin dimer (α*E-D*), and αT-catenin (α*T*). Fractions were collected from 5–20% sucrose gradients and analyzed by Coomassie-stained SDS-PAGE (*top*). The percentage of protein in each fraction was measured and plotted, and the data were fit to a Gaussian curve. *F*, sedimentation coefficient of αE-catenin dimer, αE-catenin monomer, and αT-catenin. A standard curve was created by plotting the sedimentation coefficient (S) *versus* the average sucrose gradient fraction of protein standards (similar standards as *D*; carbonic anhydrase, 2.8S; BSA, 4.3S; alcohol dehydrogenase, 7.4S; β-amylase, 8.9S; and apoferritin, 16.6S). αE-Catenin dimer, αE-catenin monomer, and αT-catenin S values were determined from the standard curve. *G*, calculated molecular masses of αE-catenin dimer, αE-catenin monomer, and αT-catenin. *H*, native PAGE analysis of recombinant αE-catenin dimer (α*Ecat D*), αE-catenin monomer (α*Ecat M*), and αT-catenin (α*Tcat*). *IEC*, ion exchange chromatography; *A.U.*, arbitrary units.

We assessed the oligomerization state of αT-catenin by chromatography. Recombinant αT-catenin protein prepared from *E. coli* was first purified by Mono Q ion exchange chromatography (Fig. 2*A*). Two peaks were routinely observed during elution off a Mono Q column (Fig. 2*A*, *top* chromatogram), and SDS-PAGE analysis of peak fractions revealed they both contained full-length αT-catenin (Fig. 2*A*, *bottom* gel). A similar ion exchange chromatography profile is observed with *M. musculus* αE-catenin (data not shown), and the two peaks correspond to the monomer (peak 1) and homodimer (peak 2) species. Both αT-catenin peak fractions were subsequently purified over a Superdex 200 (S200) size exclusion chromatography (SEC) column. The Mono Q peak 1 fraction eluted in a single, discrete peak (Fig. 2*B*, *purple line*), consistent with it being a single, likely monomeric, species. The S200 elution profile of Mono Q peak 2 was similar to peak 1, although a second, small peak was sometimes observed where a dimer species would be expected to elute (Fig. 2*B*, *red line*).

We then compared the primary S200 peak (elution volume, 60–70 ml; concentrated to 25–50 μm) of αT-catenin with αE-catenin monomer and homodimer by analytical SEC. At all concentrations tested (25–50 μm), αT-catenin eluted in a single peak after both αE-catenin homodimer and monomer, suggesting that αT-catenin is a monomer (Fig. 2*C*). We then used SEC and sucrose density gradient centrifugation to determine the molecular mass of αT-catenin, αE-catenin monomer, and αE-catenin homodimer (34). The SEC elution profiles (Fig. 2*C*) were compared with known standard proteins to calculate the Stokes radius (Fig. 2*D*). The calculated Stokes radius of αE-catenin homodimer was similar to past observations (6.5 *versus* 7.4 nm; Ref. 35), and the Stokes radii of both αE-catenin monomer and homodimer species were comparable with our previously measured radii of gyration from small angle x-ray scattering (4.5 and 6.0 nm, respectively; Ref. 32). The Stokes radius of αT-catenin was calculated to be 4.7 nm, slightly smaller than that of αE-catenin monomer (Fig. 2*D*).

We then used sucrose density gradient centrifugation to determine the sedimentation coefficients of αT-catenin, αE-catenin monomer, and αE-catenin homodimer. Proteins were separated on 5–20% sucrose gradients, and the fraction peak was determined and compared with a standard curve to calculate the sedimentation coefficient (Fig. 2, *E* and *F*). The Svedberg coefficients were determined to be 7.0S for αE-catenin homodimer (identical to past calculation (35)), 5.2S for αE-catenin monomer, and 5.7S for αT-catenin. Molecular masses were then estimated based on the measured Stokes radii and sedimentation coefficients (Fig. 2*G*). The molecular mass of αT-catenin was calculated to be 109 kDa, similar to that of αE-catenin monomer (106 kDa). Finally, αT-catenin migrated as a single band by native PAGE, faster than either αE-catenin monomer or dimer, consistent with the SEC analysis (Fig. 2*H*). We conclude that αT-catenin is primarily a monomer in solution.

Dimerization kinetics differ significantly between mouse αE-catenin and αN-catenin at physiological temperatures (8). αE-Catenin homodimerization is significantly weaker than αN-catenin homodimerization, but a kinetic block limits disassociation once an αE-catenin dimer is formed. The presence of two peaks in the Mono Q elution profile (Fig. 2*A*) and the minor peak in the peak 2 SEC elution (Fig. 2*B*) suggest that αT-catenin might exist as a homodimer. However, if the Mono Q peak 2 elution represented a homodimer species of αT-catenin, then the majority of these dimers dissociated during SEC (Fig. 2*B*). We were never able to purify a sufficient quantity of the potential dimer species for analysis by SEC or native-PAGE. Also, attempts to promote dimerization by incubation of the monomer at physiological (37 °C) temperatures caused the protein to aggregate and fall out of solution. Although we were unable to analyze the dimerization kinetics of wild-type (WT) αT-catenin, our analysis of the V94D mutant revealed that αT-catenin, similar to αE-catenin and αN-catenin, has dimerization potential (described below). Nonetheless, we took advantage of the lack of a stable dimer in solution to study the behavior of αT-catenin monomer binding to F-actin.

##### αT-Catenin Monomer Binds F-actin

**FIGURE 3.**
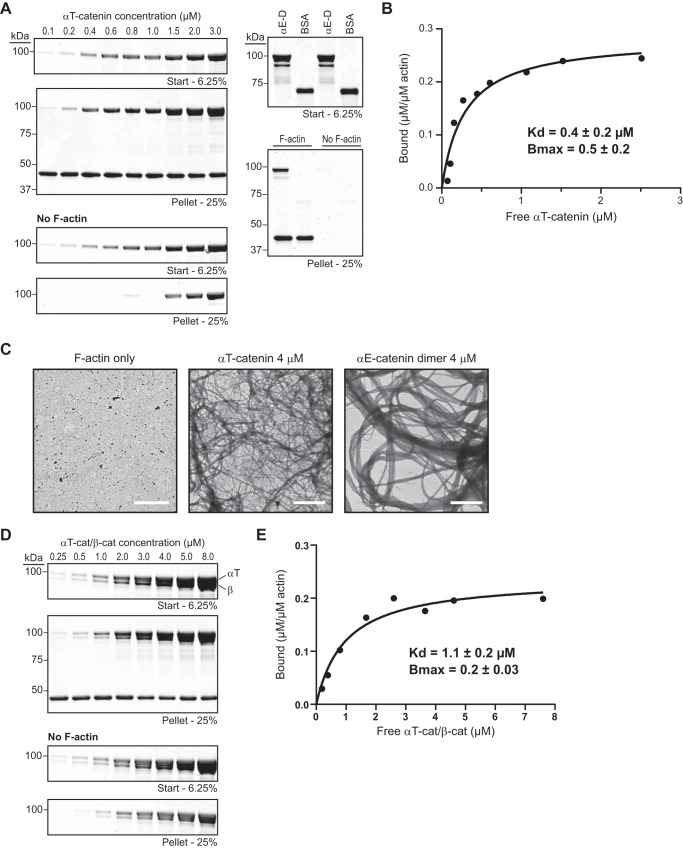
**αT-Catenin binds F-actin.** *A*, high speed cosedimentation assay of αT-catenin with F-actin. *Left panels*, increasing concentrations (0.1–3.0 μm) of αT-catenin with or without 2 μm F-actin were incubated for 30 min at room temperature and then centrifuged. Starting (6.25% of total) and pelleted materials (25% of total) were separated by SDS-PAGE and stained with Coomassie dye. *Right panels*, 4 μm αE-catenin dimer (α*E-D*) and 4 μm BSA were routinely run as positive and negative controls, respectively. *B*, bound αT-catenin (μm/μm actin) from *A* was plotted against free αT-catenin (μm), and data were fit to a hyperbolic function (*black line*). The average *K_d_* and *B*_max_ ±S.D. from four independent experiments are shown. Results from these experiments were: experiment 1, *K_d_* = 0.3 μm, *B*_max_ = 0.6; experiment 2, *K_d_* = 0.8 μm, *B*_max_ = 0.7; experiment 3, *K_d_* = 0.3 μm, *B*_max_ = 0.5; experiment 4 (binding results shown in *A* and plotted in *B*), *K_d_* = 0.3 μm, *B*_max_ = 0.3. *C*, negative stain transmission electron micrographs of 2 μm F-actin in the absence or presence of 4 μm αT-catenin or 4 μm αE-catenin homodimer. *Scale bars*, 2 μm. *D*, increasing concentrations (0.25–8.0 μm) of αT-catenin (α*T-cat*)·β-catenin (β*-cat*) complex with or without 2 μm F-actin were incubated for 30 min at room temperature and then centrifuged. Starting (6.25% of total) and pelleted materials (25% of total) were separated by SDS-PAGE and stained with Coomassie dye. *E*, bound αT-catenin·β-catenin (μm/μm actin) from *D* was plotted against free αT-catenin·β-catenin (μm), and data were fit to a hyperbolic function (*black line*). The average *K_d_* and *B*_max_ ±S.D. from three independent experiments are shown. Results from these experiments were: experiment 1 (binding results shown in *D* and plotted in *E*), *K_d_* = 1.0 μm, *B*_max_ = 0.2; experiment 2, *K_d_* = 1.4 μm, *B*_max_ = 0.2; experiment 3, *K_d_* = 1.1 μm, *B*_max_ = 0.2.

Mammalian αE-catenin binds and bundles F-actin (9–12, 36), although in the absence of force, homodimerization is required to potentiate F-actin binding. We tested whether αT-catenin monomer binds F-actin using an F-actin cosedimentation assay. Increasing concentrations of αT-catenin were incubated in the presence or absence of 2 μm F-actin, the samples were centrifuged, and the resulting pellets were analyzed. αT-Catenin cosedimented with F-actin above background (Fig. 3*A*), and the bound protein was quantified and plotted over free protein to calculate the affinity of the interaction (Fig. 3*B*). Bovine serum albumin (BSA) and αE-catenin were run as negative and positive controls, respectively (Fig. 3*A*, *right panels*). Plotted data were fit to a hyperbolic function (Fig. 3*B*). αT-Catenin bound to F-actin with a *K_d_* of 0.4 ± 0.2 μm, similar to αE-catenin dimer (1.0 μm; Ref. 36). Thus, αT-catenin monomer is a constitutive actin-binding protein, and unlike αE-catenin, homodimerization is not required for strong F-actin binding in the absence of force (9, 10, 12).

To investigate whether αT-catenin monomer bundles F-actin, we used transmission electron microscopy to visualize αT-catenin incubated with actin filaments. Weak bundling of 2 μm F-actin was observed with 4 μm αT-catenin (Fig. 3*C* and quantification in Fig. 5*D*). In contrast, robust bundling of 2 μm F-actin was observed with 4 μm αE-catenin homodimer (Figs. 3*C* and 5*D*). The weak bundling observed with αT-catenin could result from either the dimer species being stabilized on the actin filament or activation of a cryptic dimerization domain as observed in the vinculin tail (37). We conclude that αT-catenin is a poor bundler of F-actin.

##### αT-Catenin Couples β-Catenin to F-actin

Binding to β-catenin weakens the affinity of αE-catenin for F-actin (9, 10). To test whether αT-catenin can bind F-actin as part of the cadherin·catenin complex, we purified mouse β-catenin and mixed it with αT-catenin. As expected, αT-catenin bound to β-catenin with a 1:1 stoichiometry (data not shown), and we isolated the β-catenin·αT-catenin complex by SEC. Increasing concentrations of the β-catenin·αT-catenin complex were incubated in the presence or absence of F-actin and centrifuged, and the pelleted material was analyzed as above. Although the β-catenin·αT-catenin complex pelleted in the absence of F-actin (Fig. 3*D*, *No F-actin panel*), we were able to calculate the affinity of the complex for F-actin. The β-catenin·αT-catenin complex bound to F-actin with a *K_d_* of 1.1 ± 0.2 μm (Fig. 3*E*). Although β-catenin lowers the affinity of αT-catenin for F-actin slightly, the interaction strength is considerably stronger than that of the *Danio rerio* β-catenin·αE-catenin complex (>10 μm) and similar to the strength of αE-catenin homodimer association with F-actin (32, 36). Thus, αT-catenin can bind both β-catenin and F-actin simultaneously to directly link the cadherin·catenin complex to the actin cytoskeleton. This is distinct from αE-catenin in which force is needed to strengthen the association between the cadherin·catenin complex and F-actin (12). Although tension may strengthen the interaction between αT-catenin and F-actin, we speculate that basal binding permits coupling between the cadherin·catenin complex and actin through αT-catenin over a range of forces.

##### αT-Catenin V94D Mutation Creates an Obligate Homodimer

**FIGURE 4.**
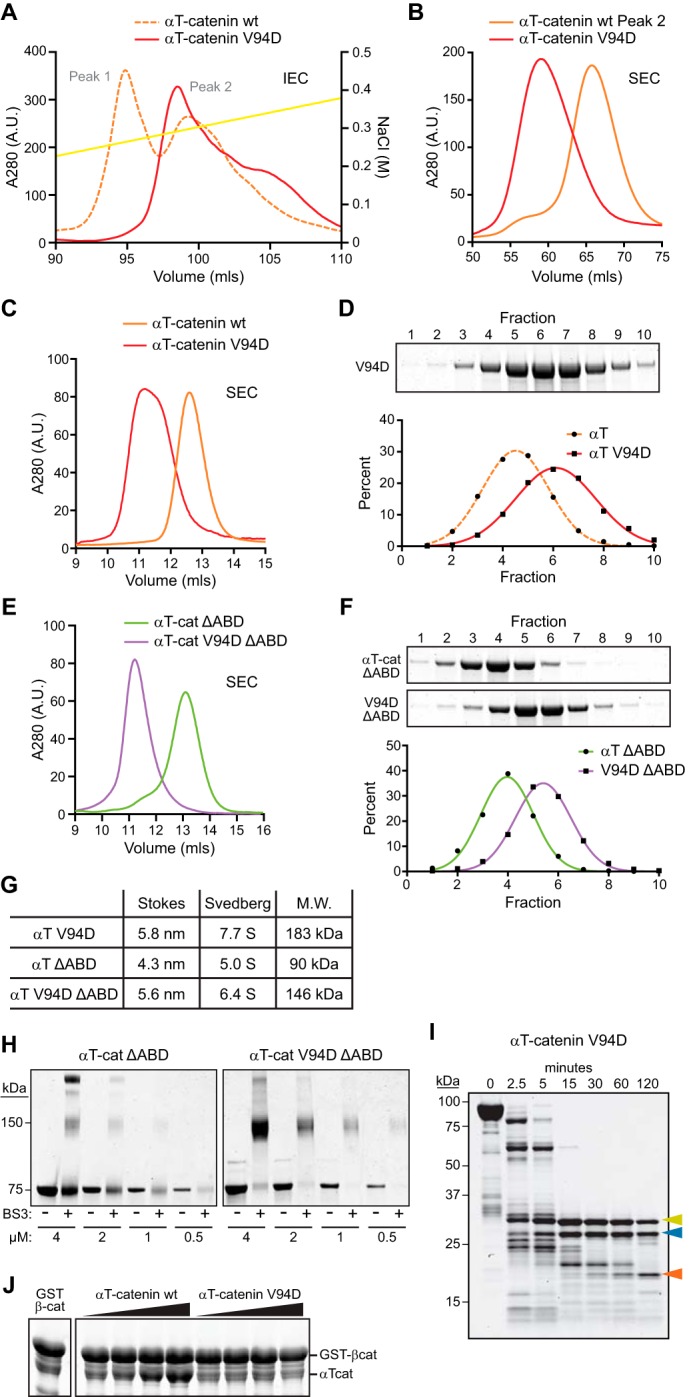
**αT-Catenin V94D mutation promotes homodimerization.** *A*, Mono Q anion exchange chromatography of αT-catenin V94D mutant (*solid red line*) and WT αT-catenin (*dashed orange line*; shown as reference; chromatogram is the same as in Fig. 2*A*). *B*, S200 SEC of αT-catenin V94D Mono Q peak fraction and αT-catenin WT peak 2 fraction. *C*, analytical S200 SEC of αT-catenin V94D and αT-catenin WT. The elution profile was used to calculate *R_S_* in *G. D*, sucrose gradient sedimentation of αT-catenin V94D. Fractions were collected from 5–20% sucrose gradients and analyzed by Coomassie-stained SDS-PAGE. The percentage of V94D in each fraction was measured and plotted, and the data were fit to a Gaussian curve (*red line*). The αT-catenin sedimentation profile from Fig. 2*E* (*dashed orange line*) is shown for comparison. The fraction peak was used to calculate the sedimentation coefficient in *G. E*, analytical S200 SEC of αT-catenin (α*T-cat*) ΔABD and αT-catenin V94D ΔABD. Elution profiles were used to calculate *R_S_* in *G. F*, sucrose gradient sedimentation of αT-catenin (α*T*) ΔABD and αT-catenin V94D ΔABD. The fraction peaks were used to calculate sedimentation coefficients in *G. G*, calculated molecular masses of αT-catenin V94D, αT-catenin ΔABD, and αT-catenin V94D ΔABD. *H*, cross-linking experiments with αT-catenin ΔABD and αT-catenin V94D ΔABD. Decreasing concentrations of protein (4-0.5 μm) were incubated with or without 1 mm BS3 for 30 min at room temperature, separated by SDS-PAGE, and stained with Coomassie dye. *I*, limited proteolysis of αT-catenin V94D. *Color-coded arrows* mark stable fragments mapped in Fig. 1*A. J*, increasing concentrations of purified αT-catenin WT or αT-catenin V94D protein were incubated with GST-tagged full-length β-catenin (β-*cat*) for 1 h at room temperature, washed, and then analyzed by SDS-PAGE. *IEC*, ion exchange chromatography; *A.U.*, arbitrary units.

Two mutations in αT-catenin have been linked to ARVC: replacement of a valine for an aspartic acid at aa 94 (V94D) in the N1 domain and deletion of a leucine at aa 765 (L765del) in the ABD (26). Yeast two-hybrid and overexpression studies suggest that the V94D mutant interferes with β-catenin binding and that the L765del mutation promotes oligomerization (26). However, it is not clear how these mutations affect the biochemical properties of αT-catenin or impact cellular function in cardiomyocytes. We used site-directed mutagenesis to make the V94D and L765del mutations in αT-catenin and attempted to purify the mutant proteins. We were unable to purify L765del; the mutation rendered the expressed protein insoluble (data not shown). However, we were successful in expressing and purifying the V94D mutant. Surprisingly, V94D eluted as a single peak off the Mono Q column rather than two as observed with WT αT-catenin (Fig. 4*A*). We then ran the V94D peak over an S200 SEC column where it eluted as a single peak before WT αT-catenin and similar to the possible homodimer peak (Fig. 4*B*). We then compared the concentrated V94D protein (25–30 μm; concentrations greater than this precipitated out of solution) with WT αT-catenin by analytical SEC. The V94D mutant eluted as a single species before WT αT-catenin with a larger Stokes radius (Fig. 4, *C* and *G*; 5.8 *versus* 4.7 nm). The V94D mutant also displayed a higher sedimentation coefficient than WT αT-catenin (Fig. 4, *D* and *G*; 7.7S *versus* 5.7S). The Stokes radius and sedimentation coefficient produced a molecular mass of 183 kDa (Fig. 4*G*), roughly double that of WT αT-catenin. We conclude that the V94D mutation creates a stable αT-catenin homodimer.

Because full-length αT-catenin V94D is difficult to purify, we deleted the ABD (aa 660–895) in both WT and V94D αT-catenin to improve protein yield. We analyzed the SEC and sedimentation properties of the ΔABD constructs (Fig. 4, *E–G*). Similar to the full-length construct, the V94D mutation altered the elution and sedimentation profiles of the ΔABD construct (Fig. 4, *E* and *F*). The calculated molecular mass of αT-catenin V94D ΔABD was 146 kDa compared with 90 kDa for αT-catenin ΔABD, consistent with it forming a homodimer.

We analyzed the oligomeric state of the αT-catenin ΔABD proteins by cross-linking. Increasing concentrations of αT-catenin ΔABD and αT-catenin V94D ΔABD were incubated with or without the cross-linker bis(sulfosuccinimidyl)suberate (BS3), and the resulting products were analyzed by SDS-PAGE. As expected, αT-catenin ΔABD and αT-catenin V94D ΔABD ran as 75-kDa proteins in the absence of cross-linker (Fig. 4*H*). In the presence of BS3, however, V94D migrated as a 150-kDa protein at all concentrations tested, indicating a cross-linked dimer. Incubation with BS3 did not affect αT-catenin ΔABD migration at low concentrations, although at higher concentrations (2 and 4 μm), a 150-kDa species was detected. We speculate that this could reflect a transient homodimer species. We conclude that the V94D mutation promotes dimerization of αT-catenin.

We used limited proteolysis to determine whether the V94D mutation affected domain organization. Like WT αT-catenin, three fragments were resistant to trypsin cleavage in V94D (Fig. 4*I*). However, the β-catenin/homodimerization domain (aa 108 start; confirmed by Edman degradation sequencing) was protected relative to WT (Fig. 4*I*, *blue arrowhead*, compare with Fig. 1*D*, *blue arrowhead*), consistent with this domain being stabilized in the homodimer state.

We then questioned whether the V94D homodimer could interact with β-catenin. We mixed increasing concentrations of WT or V94D αT-catenin with GST-tagged β-catenin, pulled down the β-catenin, and assessed binding. Wild-type αT-catenin bound GST-β-catenin at stoichiometric levels; however, little to no V94D bound (Fig. 4*J*). Thus, the V94D mutation creates an obligate αT-catenin homodimer that cannot bind β-catenin.

##### αT-Catenin V94D Binds and Bundles F-actin

**FIGURE 5.**
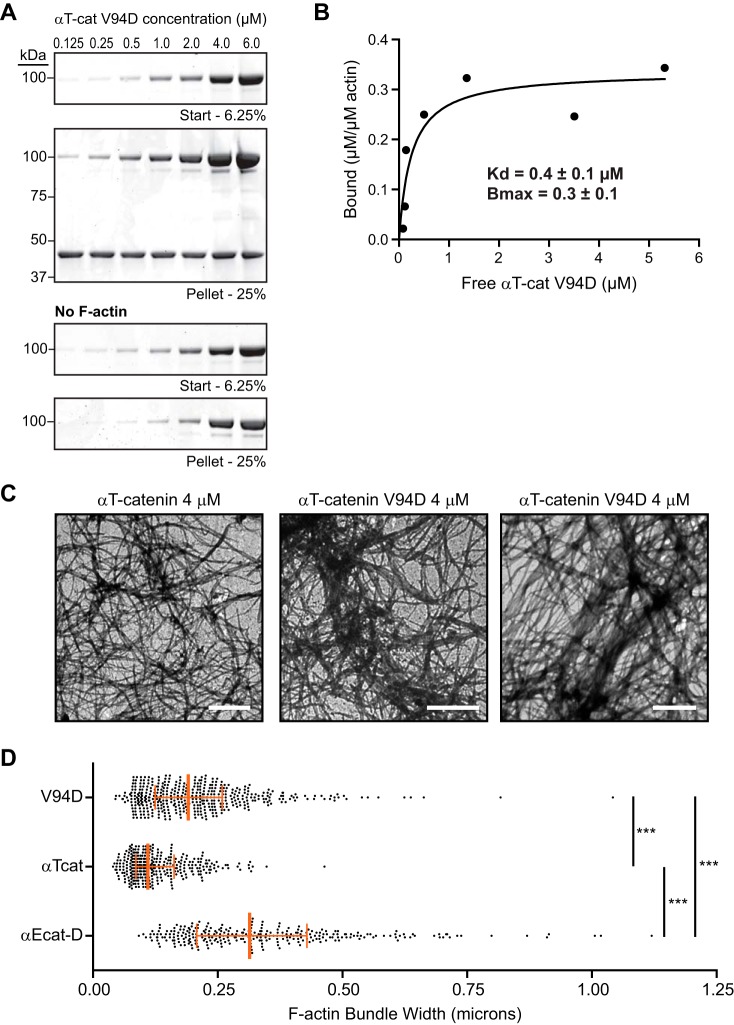
**αT-Catenin V94D binds F-actin and promotes bundling.** *A*, high speed cosedimentation assay of αT-catenin V94D with F-actin. Increasing concentrations (0.125–6.0 μm) of αT-catenin V94D with or without 2 μm F-actin were incubated for 30 min at room temperature and then centrifuged. Starting (6.25% of total) and pelleted materials (25% of total) were separated by SDS-PAGE and stained with Coomassie dye. *B*, bound αT-catenin V94D (μm/μm actin) from *A* was plotted against free αT-catenin V94D (μm), and data were fit to a hyperbolic function (*black line*). The average *K_d_* and *B*_max_ ±S.D. from three independent experiments are shown. Results from these experiments were: experiment 1, *K_d_* = 0.4 μm, *B*_max_ = 0.4; experiment 2 (binding results shown in *A* and plotted in *B*), *K_d_* = 0.2 μm, *B*_max_ = 0.3; experiment 3, *K_d_* = 0.4 μm, *B*_max_ = 0.2. *C*, negative stain transmission electron micrographs of 2 μm F-actin in the presence of 4 μm αT-catenin or 4 μm αT-catenin V94D. *Scale bars*, 2 μm. *D*, F-actin bundle width was measured in αE-catenin homodimer (α*Ecat-D*; Fig. 3*C*), WT αT-catenin (α*Tcat*), and V94D αT-catenin (*V94D*) samples. A scatter plot of all measurements (αE-catenin homodimer, *n* = 291; WT αT-catenin, *n* = 337; V94D αT-catenin, *n* = 449) from at least three images is shown. The *orange vertical line* marks the median, and the *bars* define the interquartile range. Mean and S.D. were: αE-catenin homodimer, 0.34 ± 0.17; WT αT-catenin, 0.13 ± 0.06; V94D αT-catenin, 0.21 ± 0.12). ***, *p* < 0.001, one-way analysis of variance with Dunn's multiple comparison test.

Dimerization promotes αE-catenin binding to F-actin (9, 10). We questioned whether the V94D mutation potentiates αT-catenin binding to F-actin. The V94D mutant cosedimented with F-actin (Fig. 5*A*) with an affinity similar to that of WT αT-catenin (0.4 ± 0.1 μm; Fig. 4*B*), suggesting that homodimerization does not increase the affinity of αT-catenin for F-actin.

We then tested whether the V94D homodimer could bundle F-actin. We consistently observed increased bundling of 2 μm F-actin with 4 μm αT-catenin V94D relative to 4 μm WT αT-catenin (Fig. 5, *C* and *D*). Although increased, the level of bundling was still less than that observed with 4 μm αE-catenin homodimer (Figs. 3*C* and 5*D*). We conclude that the V94D mutation promotes αT-catenin-mediated F-actin bundling.

##### αT-Catenin V94D Disrupts Localization in Cardiomyocytes

**FIGURE 6.**
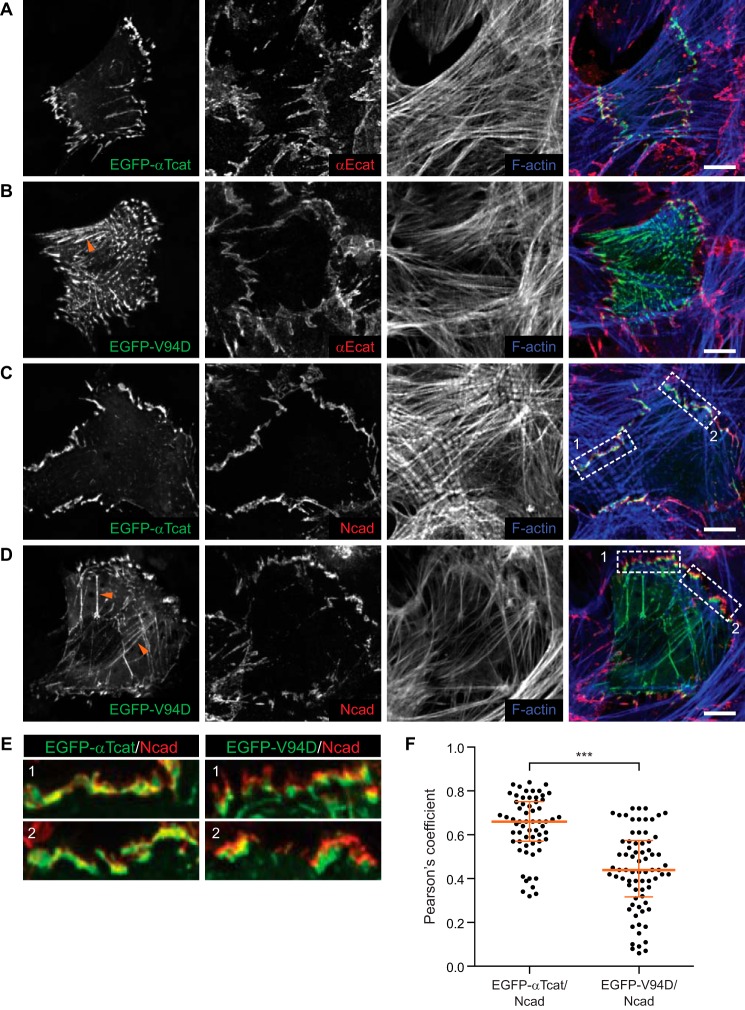
**αT-Catenin V94D mutation disrupts localization in cardiomyocytes.** *A–E*, mouse neonatal cardiomyocytes transfected with EGFP-tagged αT-catenin or αT-catenin V94D. Cells were fixed 48 h post-transfection and stained with Alexa Fluor-labeled phalloidin and antibodies against αE-catenin (*A* and *B*) or N-cadherin (*C–E*). EGFP-αT-catenin colocalized with N-cadherin at cell-cell contacts (*C*; magnification of *boxed* contacts in *E*, *left panels*), whereas EGFP-αT-catenin V94D localization was largely adjacent to N-cadherin-rich contacts (*D*; magnification of *boxed* contacts in *E*, *right panels*). EGFP-αT-catenin V94D was also observed on actin fibers (*B* and *D*, *orange arrows*). *Scale bars*, 10 μm. *F*, Pearson's *r* was calculated between N-cadherin and EGFP-αT-catenin or EGFP-αT-catenin V94D signals at individual AJ clusters in transfected cells (EGFP-αT-catenin (*EGFP-*α*Tcat*)/N-cadherin (*Ncad*), *n* = 61 AJ clusters; EGFP-αT-catenin V94D (*EGFP-V94D*)/N-cadherin, *n* = 74 AJ clusters). A scatter plot with all data points is shown. The *orange horizontal line* marks the median, and the *bars* define the interquartile range. Mean and S.D. were: EGFP-αT-catenin/N-cadherin, 0.64 ± 0.14; EGFP-αT-catenin V94D/N-cadherin, 0.44 ± 0.18. ***, *p* < 0.0001, unpaired *t* test.

αT-Catenin localizes to the adherens junction at the ICD in cardiomyocytes (22). To determine whether the V94D mutation disrupted αT-catenin cellular localization, we transiently expressed EGFP-tagged WT or V94D αT-catenin in neonatal mouse cardiomyocytes. EGFP-αT-catenin localized specifically to cell-cell contacts in cardiomyocytes where it colocalized with both αE-catenin and N-cadherin (Fig. 6, *A*, *C*, and zoom in *E*). In contrast, V94D was largely peripheral to cell-cell contacts (Fig. 6, *B*, *D*, and zoom in *E*) and localized to actin fibers (Fig. 6, *B* and *D*, *orange arrowheads*). This was confirmed by directly measuring colocalization between N-cadherin and EGFP-αT-catenin or EGFP-αT-catenin V94D signals at AJ clusters in transfected cells using Pearson's *r* (Fig. 6*F*). This analysis revealed a significant reduction in colocalization between N-cadherin and EGFP-αT-catenin V94D at AJs (Fig. 6*F*). Thus, the V94D mutation disrupts αT-catenin subcellular localization in cardiomyocytes.

## Discussion

### 

#### 

##### αT-Catenin Binds F-actin Strongly as a Monomer

Our *in vitro* results show that, in solution, αT-catenin binds F-actin as a monomer and in complex with β-catenin, properties that separate it from mammalian αE-catenin. αT-Catenin monomer binds F-actin with a slightly higher affinity than αE-catenin homodimer (0.4 *versus* 1.0 μm) (36). Although β-catenin binding reduces the affinity of αT-catenin for F-actin, the reduction is relatively small (from 0.4 to 1.1 μm). We conclude that αT-catenin binding to F-actin, unlike mammalian αE-catenin, is not allosterically regulated. This would permit αT-catenin to directly couple the cadherin·catenin complex to the actin cytoskeleton in the absence of tension, although mechanical force could strengthen the αT-catenin-actin interface.

##### αT-Catenin Has Dimerization Potential

Both *M. musculus* αE-catenin and αN-catenin homodimerize in solution, although the kinetics of dimerization differ significantly between the two mammalian α-catenins (8). At physiological temperature, the homodimerization affinity of αN-catenin is more than 10× greater than the homodimerization affinity of αE-catenin (2 *versus* 25 μm). However, the kinetics of dissociation differ markedly: αN-catenin equilibrates quickly, whereas a kinetic block limits αE-catenin dissociation (8). The αE-catenin dimer is thus stabilized and can persist at concentrations well below the *K_d_* of association. Our *in vitro* results suggest that αT-catenin has the ability to homodimerize. We observed a monomer and putative dimer species by ion exchange chromatography, although the dimer quickly dissociated upon dilution during SEC. Stronger evidence comes from our analysis of the V94D mutation where a single amino acid change shifted the protein to the homodimer state. Cross-linking studies with the αT-catenin ΔABD constructs also provide evidence for dimerization potential in the WT protein. Unfortunately, our inability to maintain soluble αT-catenin at or near physiological temperature (37 °C) precluded a detailed analysis of dimerization kinetics. Nonetheless, our results lead us to postulate that αT-catenin has dimerization potential and that the homodimer species, similar to αN-catenin, dissociates quickly (*i.e.* no kinetic block).

Evidence suggests a potential role for the α-catenin homodimer in migration and cell-cell adhesion (36, 38, 39). However, a physiological role for the α-catenin homodimer in cardiomyocytes and whether putative αE-catenin and αT-catenin homodimers function similarly *in vivo* are unclear. The V94D mutation, which drives αT-catenin into the dimer state *in vitro*, shifted localization from cell-cell contacts and promoted recruitment to F-actin bundles when expressed in cardiomyocytes. Actin filament cross-linking is essential for cardiomyocyte cytoskeletal organization and function. The barbed ends of actin filaments from adjoining sarcomeres interdigitate at the Z-disc where they are cross-linked primarily by α-actinin to form a structural lattice (40). α-Actinin is an established α-catenin ligand (41, 42), and we have detected αT-catenin in complex with α-actinin in cardiomyocyte lysates.^3^ Thus, the αT-catenin homodimer could have a role in cytoskeleton organization in cardiomyocytes. Alternatively, homodimerization may serve to regulate interactions with β-catenin and/or plakoglobin along the ICD. Additional work is needed to elucidate the putative role of the αT-catenin homodimer in cardiomyocyte biology.

##### V94D Mutation Linked to ARVC Promotes Homodimerization

The V94D mutation in αT-catenin is linked to ARVC, although the heterozygous mutation has only been documented in one individual (26). It was shown previously that the mutation reduced both β-catenin binding and homodimerization potential in a yeast two-hybrid assay (26). In contrast, we found that V94D promotes αT-catenin homodimerization, in effect creating an obligate homodimer species that cannot bind β-catenin. Not surprisingly, the V94D mutant disrupted cell-cell contact localization when expressed in cardiomyocytes. In the heterozygous state, it is unclear whether 1) V94D interacts with WT αT-catenin to disrupt localization to cell junctions and αT-catenin-mediated adhesion and/or 2) the mislocalized mutant protein disrupts cytoskeletal organization. Nonetheless, to the best of our knowledge, this is one of the first demonstrations of how a disease-linked mutation in α-catenin disrupts a fundamental molecular property.

##### αT-Catenin Domain Stability

Our limited proteolysis experiments revealed that both the β-catenin/homodimerization domain and middle domain were more protease-sensitive in αT-catenin than in αE-catenin. Notably, the N2 bundle within the β-catenin/homodimerization domain of αT-catenin is the region with the least conservation compared with αE-catenin. αT-Catenin binds β-catenin (Fig. 4*D*) and plakoglobin,^4^ although the strengths of these interactions are untested. Differences in N2 could impact αT-catenin ligand binding, including self-association, to regulate molecular complex formation at cell-cell contacts.

The core M region (M1–M3) of αE-catenin is required for its function as a mechanosensor in which tension alters α-catenin conformation to promote ligand binding (14, 16, 29, 43). Recent structural and single molecule studies coupled with molecular dynamics simulations support a model in which mechanical force reorients M2 and M3 to release M1, which contains the vinculin-binding domain (16, 17, 29). A salt bridge network between M domains is postulated to maintain αE-catenin in the autoinhibited conformation in the absence of tension (17). Based on sequence homology, a similar salt bridge network could exist in αT-catenin, although our limited proteolysis results showed that the αT-catenin M fragment (M2-M3) was less stable than in αE-catenin. We speculate that increased flexibility within the αT-catenin M2 and M3 domains could reduce the force required for activation, permitting M1 release and ligand recruitment at lower tension states.

Increased flexibility between the M2 and M3 domains could also promote ligand binding within this region. Notably, αT-catenin, but not αE-catenin, was shown to bind plakophilin-2, a desmosomal protein that links to intermediate filaments, and the binding interface was mapped to M3 (23). αT-Catenin, through association with plakophilin-2, may function as a molecular link to integrate the actin and intermediate filament cytoskeletons at the ICD. It is possible that structural differences within the core M region between α-catenins could regulate both mechanosensing and ligand binding properties.

##### αT-Catenin Function in Cardiomyocytes

α-Catenin functions in adhesion and mechanical signaling must be integrated in all tissues. Contractile forces place physical demands on heart junctional complexes: not only must they withstand repeated cycles of force but tension-sensing proteins within these complexes must be tuned to regulate signaling and maintain homeostasis. Our *in vitro* studies showed that αT-catenin could directly couple the actin cytoskeleton to cadherin·catenin in the absence of tension. We speculate that this property of αT-catenin might permit the cadherin·catenin complex to maintain a static linkage to the actomyosin network over a range of forces such as those produced by repeated cycles of contraction and relaxation in cardiomyocytes. Our biochemical analyses also suggest that αT-catenin dimerization properties and M region stability differ from those in αE-catenin. How these differences impact *in vivo* function is unclear, but we speculate that they could impact molecular interactions and tension sensing. In the mammalian heart, αT-catenin may have evolved to complement αE-catenin functions in adhesion and signaling.

## Experimental Procedures

### 

#### 

##### Plasmids

DNA encoding full-length *M. musculus* αT-catenin was cloned into pGEX-TEV (36) to create a fusion between GST and αT-catenin. Site-directed mutagenesis was used to create the valine to aspartic acid mutation at amino acid 94 (V94D) in αT-catenin. The N-terminal head region (aa 1–659) of αT-catenin or αT-catenin V94D was cloned into pGEX-TEV to create the ΔABD constructs. WT and V94D αT-catenin were cloned into pEGFP-C1 for expression in mammalian cells.

##### Recombinant Protein Expression and Purification

GST-tagged αT-catenin, αE-catenin, and β-catenin were expressed in BL21(DE3) *E. coli* cells and purified as described (31, 36). GST-tagged proteins bound to glutathione-agarose were equilibrated in cleavage/elution buffer (20 mm Tris, pH 8.0, 150 mm NaCl, 2 mm EDTA, 1 mm DTT, and 10% glycerol) and then incubated with tobacco etch virus protease overnight at 4 °C to cleave protein from the GST tag. All proteins were purified by Mono Q anion exchange chromatography followed by S200 gel filtration chromatography in 20 mm Tris, pH 8.0, 150 mm NaCl, 10% glycerol, and 1 mm DTT. Eluted protein was concentrated to 20–50 μm working concentrations using a Millipore column concentrator, flash frozen in liquid nitrogen, and stored at −80 °C.

##### Size Exclusion Chromatography

Analytical SEC was performed at 4 °C on a Superdex 200 column in 20 mm Tris, pH 8.0, 150 mm NaCl, and 1 mm DTT. Protein was injected at 25–30 μm.

##### Native PAGE

FPLC-purified αE-catenin and αT-catenin were diluted in cold native gel sample buffer (20 mm Tris, pH 6.8, 150 mm NaCl, 300 mm sucrose, 100 mm DTT, and 0.02% bromphenol blue) and loaded onto a 5% native gel (running gel, 0.4 m Tris, pH 8.8, and 5% acrylamide; stacking gel, 0.1 m Tris, pH 6.8, and 5% acrylamide). Gels were run at 80 V for 5 h at 4 °C, stained with Coomassie Blue, and imaged on a LI-COR Biosciences scanner.

##### Limited Proteolysis and Edman Degradation Sequencing

12 μm αT-catenin was incubated at room temperature in 0.05 mg/ml sequencing grade trypsin (Roche Applied Science) in 20 mm Tris, pH 8.0, 150 mm NaCl, and 1 mm DTT. Reactions were stopped with 2× Laemmli buffer at the indicated times, and samples were analyzed by SDS-PAGE. For N-terminal sequencing, digested peptides were blotted onto PVDF membrane; stained with 0.1% Coomassie Blue R-250, 40% methanol, and 1% acetic acid; destained; and dried. Individual bands were excised and sequenced by Edman degradation (Iowa State University Protein Facility).

##### Stokes Radius Measurements

The Stokes radius (*R_S_*) was determined by analytical size exclusion chromatography using a Superdex 200 column equilibrated with 20 mm Tris, pH 8.0, 150 mm NaCl, and 1 mm DTT. Standard proteins were bovine carbonic anhydrase (*R_S_* = 2.4 nm), bovine serum albumin (*R_S_* = 3.5 nm), yeast alcohol dehydrogenase (*R_S_* = 4.6 nm), sweet potato β-amylase (*R_S_* = 5.4 nm), horse spleen apoferritin (*R_S_* = 6.7 nm), and bovine thyroglobulin (*R_S_* = 8.5 nm). The partition coefficient, *K*_av_, was calculated for all standards and α-catenin proteins used in this study. The Stokes radius was calculated from a standard curve of (−log*K*_av_)^1/2^ *versus R_S_*.

##### Sucrose Density Gradient Centrifugation

Gradients of sucrose were made by layering sucrose dissolved in 20 mm Tris, pH 8.0, and 150 mm NaCl from 20 to 5% in 2.5% increments in 13 × 63-mm ultracentrifuge tubes as described (44). Each layer was frozen in a dry ice/ethanol bath before the addition of the next layer. Tubes were stored at −80 °C until use. Tubes were thawed overnight at 4 °C to establish a gradient. 100 μl of sample was layered on top and centrifuged in a Thermo Scientific Sorvall S100-AT rotor at 70,000 rpm (200,000 × *g*) for 4 h at 4 °C. All α-catenin proteins were loaded at concentrations ≥20 μm. After centrifugation, 200-μl fractions were collected and analyzed by SDS-PAGE. Gels were imaged on a LI-COR Biosciences scanner, and the percentage of protein in each fraction was measured in ImageJ. Plotted data were fit to a Gaussian curve to determine the peak fraction in Prism software. Standard proteins were bovine carbonic anhydrase (2.8S), bovine serum albumin (4.3S), yeast alcohol dehydrogenase (7.4S), sweet potato β-amylase (8.9S), and horse spleen apoferritin (16.6S). The sedimentation coefficient of α-catenin proteins was determined from a standard curve of sedimentation coefficient (S) *versus* fraction.

##### Molecular Mass Calculations

The molecular mass of α-catenin proteins used in this study was calculated from the measured Stokes radius and sedimentation coefficient as described (34, 45).

##### Actin Cosedimentation Assays

Chicken muscle G-actin (Cytoskeleton, Inc.) was incubated in 1× actin polymerization buffer (20 mm HEPES, pH 7.5, 100 mm KCl, 2 mm MgCl_2_, 0.5 mm ATP, and 1 mm EGTA) for 1 h at room temperature to polymerize filaments. Gel-filtered αT-catenin or αT-catenin·β-catenin heterocomplex was diluted to the indicated concentrations in 1× reaction buffer (20 mm HEPES, pH 7.5, 150 mm NaCl, 2 mm MgCl_2_, 0.5 mm ATP, 1 mm EGTA, 1 mm DTT, and 0.02% Thesit) with and without 2 μm F-actin and incubated for 30 min at room temperature. Samples were centrifuged at 50,000 rpm (>100,000 × *g*) for 20 min at 4 °C in an S100-AT3 rotor. Pellets were resuspended in Laemmli sample buffer, separated by SDS-PAGE, and stained with Coomassie Blue. Gels were imaged on a LI-COR Biosciences scanner and measured and quantified in ImageJ. To determine the amount of bound protein, background sedimentation (no F-actin pellet) was first subtracted from cosedimentation (F-actin pellet). Bound protein across samples was then normalized to the F-actin pellet. The amount of bound protein was calculated from a standard curve created from the starting material. All binding data were processed with Prism software.

##### F-actin Bundling

Protein samples were prepared as for the actin cosedimentation assays and deposited on carbon grids. Samples were fixed in 2.5% glutaraldehyde, stained with 1% uranyl acetate for 1–3 min, and examined in a JEOL JEM-1011 transmission electron microscope. To quantify bundling, a 20 × 20-μm grid was overlaid on images, and the width of all bundles in four random squares on the grid was measured using ImageJ. The data were plotted and analyzed with Prism software.

##### Cross-linking Experiments

Purified αT-catenin ΔABD and αT-catenin V94D ΔABD were incubated with or without 1 mm BS3 (Thermo Scientific) in 20 mm HEPES, pH 7.4, 150 mm NaCl, and 1 mm DTT for 30 min at room temperature, separated by SDS-PAGE, stained with Coomassie dye, and imaged on a LI-COR Biosciences scanner.

##### GST Pulldown Experiments

Increasing amounts of αT-catenin or αT-catenin V94D (1–15 μg) were added to 15 μg of GST-β-catenin bound to glutathione-agarose in 20 mm Tris, pH 8, 150 mm NaCl, and 5 mm DTT. Samples were incubated with gentle mixing for >2 h at 4 °C and then washed five times in PBS + 0.05% Tween 20 and 5 mm DTT before elution in Laemmli sample buffer. Samples were separated by SDS-PAGE, stained with Coomassie dye, and imaged on a LI-COR Biosciences scanner.

##### Cardiomyocyte Isolation and Culture

All animal work was approved by the University of Pittsburgh Division of Laboratory Animal Resources. Cardiomyocytes were isolated from mouse neonates (P1–P3) as described (46). Cardiomyocytes were plated onto collagen-coated coverslips and maintained in 78% DMEM, 17% M-199, 4% horse serum, 1% penicillin/streptomycin, 1 μm Ara-C, and 1 μm isoproterenol. Transfections were performed 24 h postplating using Lipofectamine 2000 (Life Technologies).

##### Immunostaining and Confocal Microscopy

Cells were fixed in 4% paraformaldehyde in PHEM buffer (60 mm 1,4-piperazinediethanesulfonic acid, pH 7.0, 25 mm HEPES, pH 7.0, 10 mm EGTA, pH 8.0, 2 mm MgCl_2_, and 0.12 m sucrose), washed with PBS, blocked for 1 h at room temperature in PBS + 10% BSA, washed three times in PBS, incubated with primary in PBS + 1% BSA for 1 h at room temperature, washed three times in PBS, incubated with secondary in PBS + 1% BSA for 1 h at room temperature, washed three times in PBS, and mounted in Fluoromount G (Electron Microscopy Sciences). F-actin was stained using Alexa Fluor-phalloidin (Invitrogen) and antibodies against αE-catenin (Enzo Life Sciences) or N-cadherin (Invitrogen). Cells were imaged on a Nikon Eclipse Ti inverted microscope outfitted with a Prairie swept field confocal scanner, Agilent monolithic laser launch, and Andor iXon3 camera using NIS-Elements imaging software. Maximum projections of 4-μm image stacks were created for image analysis and presentation. For Pearson's *r* calculations, signal colocalization was measured between user-defined N-cadherin-positive AJ clusters and EGFP signals using ImageJ. Colocalization data were plotted and analyzed with Prism software.

## Author Contributions

A. V. K. conceived and coordinated the study and designed experiments. E. D. W., I. W. D., C. D. M., J. A. H., D. B. S., and A. V. K. performed research. E. D. W., C. D. M., J. A. H., and A. V. K. analyzed data. A. V. K. wrote the paper.

## Footnotes

3 E. D. Wickline and A. V. Kwiatkowski, unpublished observation.

4 A. V. Kwiatkowski, unpublished observation.

## Footnotes

2 The abbreviations used are:

AJ: adherens junction
F-actin: filamentous actin
ARVC: arrhythmogenic right ventricular cardiomyopathy
ICD: intercalated disc
SEC: size exclusion chromatography
ABD: actin-binding domain
T: testes
E: epithelial
M: middle
aa: amino acid(s)
N: N-terminal
S200: Superdex 200
BS3: bis(sulfosuccinimidyl)suberate
EGFP: enhanced GFP
*R_S_*: Stokes radius.
